# *NOTCH3* rs1043996 Polymorphism Is Associated with the Occurrence of Alcoholic Liver Cirrhosis Independently of *PNPLA3* and *TM6SF2* Polymorphisms

**DOI:** 10.3390/jcm10194621

**Published:** 2021-10-08

**Authors:** Ana Bainrauch, Dino Šisl, Antonio Markotić, Ana Ostojić, Slavko Gašparov, Valerija Bralić Lang, Nataša Kovačić, Danka Grčević, Anna Mrzljak, Tomislav Kelava

**Affiliations:** 1 Department of Internal Medicine, Merkur University Hospital, 10000 Zagreb, Croatia; ana.bainrauch@gmail.com; 2 Laboratory for Molecular Immunology, Croatian Institute for Brain Research, University of Zagreb, 10000 Zagreb, Croatia; dino.sisl@mef.hr (D.Š.); natasa.kovacic@mef.hr (N.K.); danka.grcevic@mef.hr (D.G.); 3 Department of Physiology and Immunology, School of Medicine, University of Zagreb, 10000 Zagreb, Croatia; 4 Department of Physiology, School of Medicine, University of Mostar, 88000 Mostar, Bosnia and Herzegovina; antoniomarkotic@msn.com; 5 Center for Clinical Pharmacology, University Clinical Hospital Mostar, 88000 Mostar, Bosnia and Herzegovina; 6 Department of Gastroenterology and Hepatology, University Hospital Center Zagreb, University of Zagreb, 10000 Zagreb, Croatia; ostojicana.zg@gmail.com; 7 School of Medicine, University of Zagreb, 10000 Zagreb, Croatia; gasprovslavko@gmail.com; 8 Department of Pathology and Cytology, Merkur University Hospital, 10000 Zagreb, Croatia; 9 Private Family Physician Office Zagreb, 10000 Zagreb, Croatia; valerija.bralic.lang@gmail.com; 10 Department of Anatomy, School of Medicine, University of Zagreb, 10000 Zagreb, Croatia

**Keywords:** single nucleotide polymorphisms, alcoholic liver disease, liver transplantation, hepatocellular carcinoma, Notch, *PNPLA3*

## Abstract

Alcoholic liver cirrhosis (ALC) is the most common indication for liver transplantation (LT) in Croatia and presents a risk factor for the development of hepatocellular carcinoma (HCC). However, genetic susceptibility has not yet been systematically studied. We aimed to investigate the contribution of the risk polymorphisms *PNPLA3* rs738409, *EGF* rs4444903, *TM6SF2* rs58542926, *MTHFR* rs1801133, previously identified in other populations and, additionally, the contribution of Notch-related polymorphisms (*NOTCH1* rs3124591, *NOTCH3* rs1043996 and rs1044116, *NOTCH4* rs422951). The study included 401 patients. The ALC group consisted of 260 LT candidates, 128 of whom had histopathologically confirmed HCC, and 132 of whom were without HCC. The control group included 141 patients without liver disease. Genotyping was performed by PCR using Taqman assays. The patients’ susceptibility to ALC was significantly associated with *PNPLA3* rs738409, *TM6SF2* rs58542926, and *NOTCH3* rs1043996 polymorphisms. These polymorphisms remained significantly associated with ALC occurrence in a logistic regression model, even after additional model adjustment for sex and age. Cirrhotic patients with the *PNPLA3* GG genotype demonstrated higher activity of ALT aminotransferases than patients with CC or CG genotypes. The susceptibility to the development of HCC in ALC was significantly associated with *PNPLA3* rs738409 and *EGF* rs4444903 polymorphisms, and logistic regression confirmed these polymorphisms as independent predictors.

## 1. Introduction

End-stage alcoholic liver disease is one of the leading indications for liver transplantation (LT), and patients with alcoholic liver cirrhosis (ALC) are at higher risk for developing hepatocellular carcinoma (HCC) [1,2,3]. Genetic predisposition contributes to both liver fibrosis and its progression to HCC. The findings of both genome-wide association studies and candidate gene approach studies, conducted in the last decade, point to the patatin-like phospholipase domain-containing protein 3 (*PNPLA3*) rs738409 G allele and the transmembrane 6 superfamily member 2 (*TM6SF2*) rs58542926 T allele as the major genetic factors that increase susceptibility to the fibrosis [4,5,6,7,8]. These two single-nucleotide polymorphisms (SNPs) are also associated with the occurrence of HCC, while methylenetetrahydrofolate reductase (*MTHFR*) rs1801133 and epidermal growth factor (*EGF*) rs4444903 may further add to the HCC risk [9,10,11,12,13]. In Southeastern Europe, ALC is the most common indication for LT, but genetic susceptibility to it has not been systematically studied.

Recent studies on profibrotic and carcinogenic genes suggest that the evolutionarily conserved Notch signaling pathway may play a critical role in hepatic fibrogenesis and carcinogenesis [14,15,16,17]. Clinical studies have shown that Notch signaling is activated in liver samples from cirrhotic or HCC patients [15,18]. In addition, preclinical studies in various murine models showed that the inhibition of Notch signaling prevents fibrogenesis, while its overexpression promotes liver tumor formation in mice [18,19,20]. Therefore, Notch-related molecules might be a promising target for the development of new antifibrotic and anticancer treatments [21,22]. Notch-related SNPs have been associated with an increased susceptibility to the development of breast cancer and poorer lung and liver cancer prognosis [23,24,25]. Still, their role as a risk factor for the development of cirrhosis or HCC has not yet been investigated.

In this study, we aimed to investigate the contribution of previously implicated genetic risk factors (*PNPLA3*, *TM6SF2*, *EGF*, *MTHFR*) for the development of cirrhosis and HCC in the Croatian population. Moreover, we introduced Notch-related SNPs to assess their potential role as risk factors. The association of the analyzed SNPs with clinical indices of disease severity was also determined as a secondary objective.

## 2. Materials and Methods

### 2.1. Study Population and Data Collection

After obtaining approval from the Merkur University Hospital and School of Medicine University of Zagreb Ethics Committee, the study included 401 patients. The ALC group consisted of 260 patients, 128 of whom had HCC, and 132 of whom were without HCC. The control group included 141 patients without liver disease. All the subjects were of Caucasian ethnicity and had no concomitant liver etiologies (viral, autoimmune or metabolic). In the ALC group, 253 patients received LT, and the presence or absence of HCC was confirmed by pathological examination of the explanted liver. In seven patients who did not undergo an LT, HCC was confirmed by radiological findings and examination of tissue obtained by liver biopsy. The data on alanine aminotransferase (ALT), aspartate aminotransferase (AST) activities, creatinine, alpha-fetoprotein (AFP) levels, tumor size, number of nodules, and microangioinvasion were obtained from the patients’ hospital clinical records.

### 2.2. DNA Isolation and Genotyping

After obtaining written informed consent to participate in the study, venous blood samples were collected and stored at −20 °C until DNA isolation. DNA was extracted from the whole blood samples (200 uL) using the QIAGEN QIAamp DNA Blood Mini Kit spin method, according to the manufacturer′s instructions. The DNA concentration and quality were determined using the NanoDrop ND1000 spectrophotometer, as described in previous studies [26]. All the samples were stored at −20 °C until the batch genotype analysis. The genotypes were determined by polymerase chain reaction (PCR) using commercially available TaqMan SNP assays for *PNPLA3* (rs738409), *EGF* (rs4444903), *TM6SF2* (rs58542926), *MTHFR* (rs1801133), *NOTCH1* (rs3124591), *NOTCH3* (rs1043996 and rs1044116), and *NOTCH4* (rs422951), using the ABI 7500 instrument (Applied Biosystems). All the SNPs were selected based on data from a haplotype map of the human genome (HapMap) with minor allele frequency (MAF) in the European population higher than 20%, except for *TM6SF2*, for which the MAF was less than 20%. However, the reason for its inclusion was its previously reported strong association with susceptibility to liver disease. The potential limitations of the results of the *TM6SF2* analysis are addressed in detail in the Discussion section. The assay IDs and expected MAFs are provided in the Supporting information (Table A1 in Appendix A).

### 2.3. Statistical Analysis

Continuous variables are presented as median with interquartile range (IQR) or mean ± standard deviation (SD) and compared using Mann-Whitney/Kruskal-Wallis test or Student’s *t*-test/ANOVA, as appropriate. Categorical data were compared using the chi-square test. The genotype frequencies of all the polymorphisms were tested for Hardy–Weinberg equilibrium. For the genotype analysis we used dominant, recessive, codominant and log additive models. For the multiple comparison correction, the minimal false discovery rate level (Storey’s q) was calculated [27]; results with both *p* < 0.05 and *q* < 0.05 were considered statistically significant. Multiple logistic regression was used to evaluate independent predictors of ALC or HCC development. The statistical analyses were performed using the free online software SNPStats (http://bioinfo.iconcologia.net/snpstats accessed on: 14 August 2021) and R (a language and environment for statistical computing, R Foundation for Statistical Computing, Vienna, Austria), with the figures plotted in GraphPad Prism version 6 for Windows (GraphPad Software Inc., La Jolla, CA, USA).

## 3. Results

### 3.1. Demographic, Laboratory and Histopathology Information

Basic demographic, laboratory, and histopathology information on the patient groups are shown in Table 1. The patients were predominantly males (89%), without any statistically significant difference in age or sex between the groups. The patients with HCC had higher AFP and ALT levels and lower creatinine levels than the patients without HCC. The mean size of the HCC was 34.15 ± 19.84 mm. Microangioinvasion was found in 37.2% of the histopathological findings.

### 3.2. Assessment of Genetic Susceptibility to Alcoholic Cirrhosis

**Figure A1:**
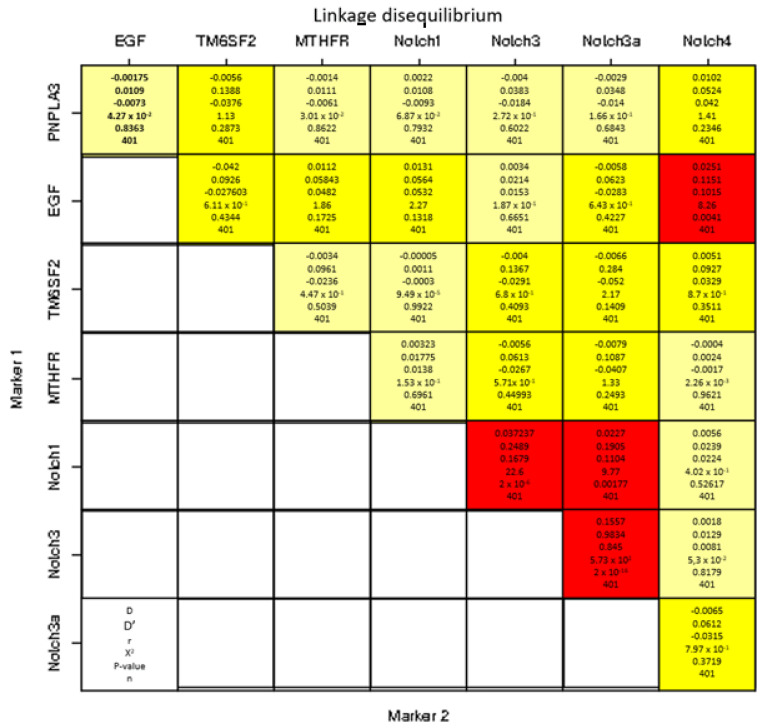
Linkage disequilibrium for the examined single-nucleotide polymorphisms. Analysis was performed using the free online software SNPStats (http://bioinfo.iconcologia.net/snpstats, accessed on: 14 August 2021). *EGF* rs4444903; *MTHFR* rs1801133; *NOTCH1* rs3124591; *NOTCH3* rs1043996; *NOTCH3*a rs1044116; *NOTCH4* rs422951; *PNPLA3* rs738409; *TM6SF26* rs58542926.

All the genotypes were in Hardy–Weinberg equilibrium (*p* > 0.05 for all SNPs examined). We observed a linkage disequilibrium between the *NOTCH3* rs1043996 and rs1044116 (r = 0.84). Weak linkage disequilibrium was present between the *NOTCH3* and *NOTCH1*, as well as between *NOTCH4* and *EGF* (Figure A1 in Appendix B).

*PNPLA3* rs738409, *TM6SF2* rs58542926 and *NOTCH3* rs1043996 were significantly associated with susceptibility to cirrhosis, whereas no association was found for the remaining five SNPs examined. In a recessive model, the presence of the *PNPLA3* GG genotype was markedly associated with a greater likelihood for liver cirrhosis development (OR 95%CI = 4.03 (1.93–8.41), *p* = 8.4 × 10^−^^5^), and significant associations were also found in dominant, codominant and log additive models (Table 2). The presence of the T allele for *TM6SF2* polymorphism was associated with greater odds of cirrhosis with OR 95%CI = 2.86 (1.56–5.23) in the dominant model (*p* = 4.4 × 10^−4^). Finally, the *NOTCH3* rs1043996 GG genotype was associated with a lower susceptibility to liver cirrhosis in a recessive model (OR 95%CI = 0.39 (0.19–0.80, *p* = 0.01). Detailed results for all the analyzed models are shown in Table 2.

The independence of predictors was further analyzed by multiple logistic regression. All three SNPs remained significantly associated with the susceptibility to cirrhosis, even after additional model adjustment for sex and age (Table 3).

#### SNPs’ Association with Pre-Transplantation Parameters

**Figure 1:**
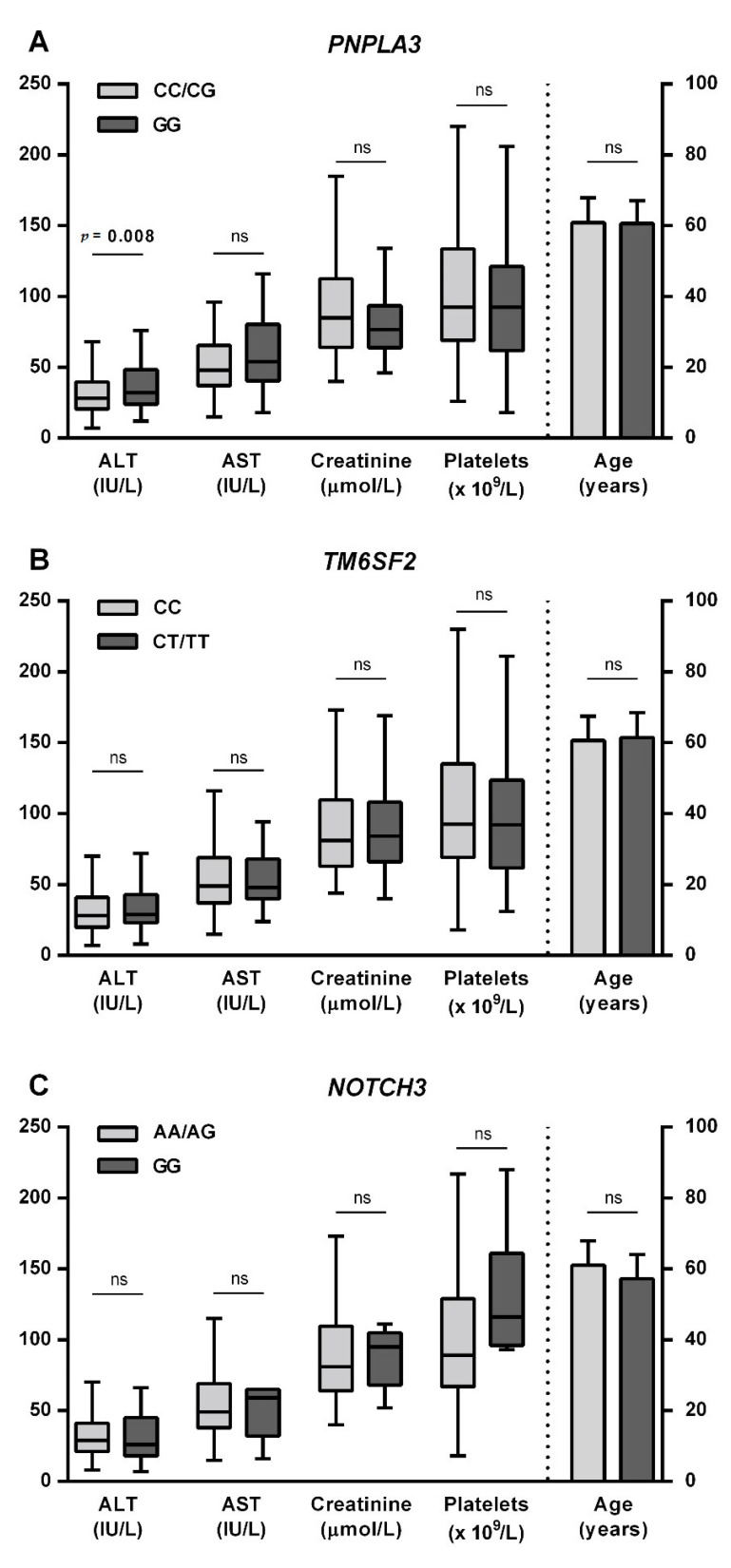
Association of SNPs with pre-transplantation parameters. Comparisons for (**A**) *PNPLA3*, (**B**) *TM6SF2*, and (**C**) *NOTCH3* rs1043996 genotypes are shown. Alanine aminotransferase (ALT), aspartate transaminase (AST), creatinine levels, and platelets were not normally distributed and are presented as median with interquartile range; the Mann–Whitney-U test was used for statistical comparison. Age at transplantation was normally distributed; therefore, data are presented as mean with standard deviation and the Student’s *t*-test was used for statistical comparison. ALT—alanine aminotransferase; AST—aspartate transaminase; ns—not significant; *PNPLA3*—patatin-like phospholipase domain-containing protein 3; *TM6SF2*—transmembrane 6 superfamily member 2.

To further establish the role of the studied SNPs in the development of ALC, we analyzed their association with pre-transplantation parameters. Patients with the *PNPLA3* GG genotype had significantly higher ALT activity in sera than patients with CC or CG genotype, while there was no difference in the creatinine concentration, platelet count or age of the patients at LT. None of the parameters differed between *TM6SF2* genotypes. We found a higher platelet count in patients with the *NOTCH3* rs1043996 GG (*p* = 0.03) genotype, but the difference did not reach statistical significance after correcting for multiple comparison, and there was no significant difference in the other parameters that were examined (Figure 1). For the remaining five examined SNPs, the analysis did not reveal any statistically significant differences (data not shown).

### 3.3. Assessment of Genetic Susceptibility to the Development of HCC in ALC Patients

The analysis showed that patients with the *PNPLA3* GG genotype are more susceptible to HCC occurrence. A significant association was found in the recessive model (OR 95%CI = 2.96 (1.57–5.58), *p* < 0.001) and in the codominant and log-additive models. The results did not show any significant associations for the other two SNPs that were significantly associated with the susceptibility to cirrhosis (*TM6SF2* rs58542926 and *NOTCH3* rs1043996). On the other hand, the SNP for *EGF*, which was not associated with a risk of cirrhosis, was significantly associated with an increased likelihood of HCC progression in the dominant model (OR 95%CI = 1.97 (1.15–3.36), *p* = 0.012, for GG/AG vs. AA genotype). No significant association with HCC was found for any other SNP (detailed results are presented in Table 4). Multiple logistic regression revealed that SNPs for the *PNPLA3* and *EGF* genes are independent predictors of the HCC development (Table 5).

#### Association of SNPs with HCC-Related Parameters

**Figure 2:**
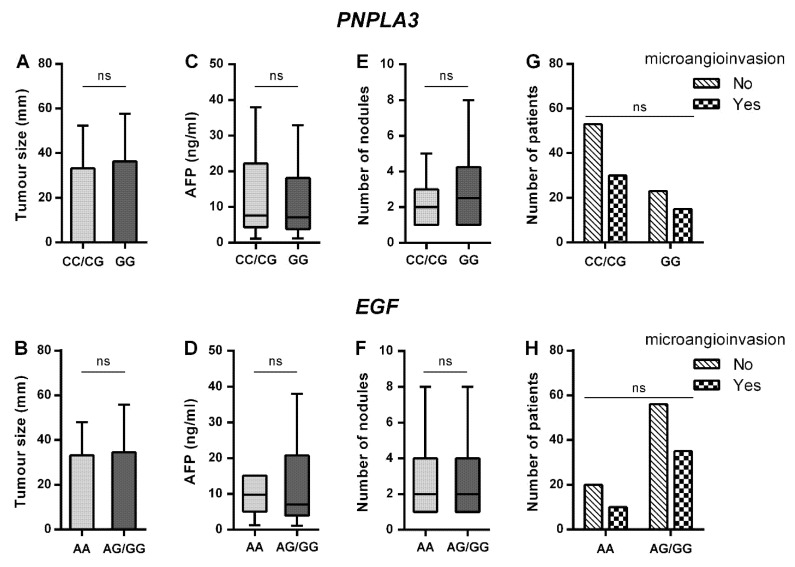
Association of SNPs with HCC-related parameters. Tumor size (**A**,**B**), alpha-fetoprotein levels (**C**,**D**), and number of nodules (**E**,**F**) are presented as median with interquartile range; the Mann–Whitney-U test was used for statistical comparison. For microangioinvasion (**G**,**H**), the number of patients is shown, and the chi-square test was used for statistical comparison. AFP—alpha-fetoprotein; *EGF*—epidermal growth factor; ns—not significant; *PNPLA3*—patatin-like phospholipase domain-containing protein 3.

There was no association between HCC-related parameters (tumor size, microangionvasion, number of nodules or AFP levels) and the SNPs of the genes significantly associated with HCC occurrence (*PNPLA3* and *EGF*, Figure 2), or between any of the other polymorphisms studied (data not shown).

## 4. Discussion

This is the first study to systematically examine the genetic susceptibility to ALC in the Southeast European population, where ALC is the leading cause of LT. A strength of this study is that it included age- and sex-matched patients who underwent LT and were diagnosed with HCC by histopathologic examination, which is superior to the radiologic diagnosis and/or mathematical correction for age usually performed in similar studies. Data from previous studies conducted in different populations showed a consistent association between *PNPLA3* and *TM6SF2* polymorphisms and susceptibility to ALC [4,5,6,7,8,28,29]. The study shows that they are also important risk factors in Croatia. To further establish the additional genetic risk factors for ALC, we investigated the possible contribution of polymorphisms in Notch receptors and found that *NOTCH3* rs1043996 GG carriers have a reduced probability of developing ALC that is independent of the *PNPLA3* or *TM6SF2* genotypes. Yu et al. [25] reported a significant association between *NOTCH3* and *NOTCH4* SNPs with HCC survival, but to the best of our knowledge, the association with ALC or HCC incidence has not been studied. Notch signaling mediates communication between neighboring cells to guide cell fate decisions both during embryogenesis and postnatal life. In the liver, Notch signaling is required for normal development, but recent findings have also identified Notch activation as an essential contributor to fibrogenesis in several murine models of fibrosis, and the overexpression of Notch has been confirmed in human samples of cirrhotic liver tissue as well as in liver cancer [15,16,18,20,30,31].

Patients with ALC have a higher risk of developing HCC, with a cumulative 5-year risk of approximately 8% [32]. The activation of the Notch signaling pathway has recently been linked to the occurrence of HCC [33]. We found an association between the *PNPLA3* and *EGF* polymorphisms and susceptibility to HCC, but no association was found for *MTHFR*, *TM6SF2*, or Notch-related polymorphisms. Tanabe et al. [34] reported that *EGF* rs4444903 is associated with HCC and found higher *EGF* secretion in carriers of the G allele. Later studies, mainly in patients with hepatitis C virus-related cirrhosis, confirmed this finding, but independence from *PNPLA3*, a dominant risk factor, was not tested [35,36]. Our results confirm *EGF* as a risk factor in ALC-related HCC and logistic regression shows its independence from *PNPLA3*. Furthermore, we show that *EGF* is not a risk factor for the development of ALC, which has not been previously investigated, to the best of our knowledge. Compared to the CC genotype, heterozygosity for *TM6SF2* (CT genotype) was clearly associated with a higher probability of developing ALC, but it was not associated with progression to HCC (OR 95%CI = 1.03 (0.58–1.83), *p* = 0.36). Due to the low incidence of the TT genotype in the *TM6SF2* gene, the study was unable to assess its association with ALC or HCC; however, four patients with this genotype had HCC, one patient had ALC, and no patients in the control group had this genotype, which suggests a potential link. Therefore, the TT genotype for *TM6SF2* may be a risk factor for a small number of patients, but its role needs to be further confirmed by a meta-analysis of a large number of studies.

Previous studies also suggested a possible role of *MTHFR* rs1801133 in HCC progression, but further research showed conflicting results. This includes two recent meta-analyses, one of which confirmed the association, while the other rejected it, with the exception of the Asian subpopulation [9,11,37]. Recently, Pineda-Tenor et al. [38] reported that *MTHFR* rs1801133 polymorphism is associated with the progression of liver fibrosis in chronic hepatitis C. However, we found no association between the *MTHFR* polymorphism and susceptibility to ALC or HCC.

In addition, we found higher activity of ALT aminotransferases in the sera of GG *PNPLA3* carriers, but there was no significant association between the *PNPLA3* genotype and the patient’s age at transplantation or HCC-related prognostic factors, such as tumor size and microangioinvasion. This is in line with the results of the study by Khlaiphuengsin et al. [39], which did not find any relationship between the *PNPLA3* genotype and tumor clinical characteristics. In addition, the results did not show a significant association between other examined SNPs and indices of disease severity.

## 5. Conclusions

This study showed that the *NOTCH3* rs1043996 GG genotype is associated with lower susceptibility to ALC, independently of *PNPLA3* and *TM6SF2* polymorphisms. The occurrence of HCC in ALC was associated with *PNPLA3* and *EGF* polymorphisms, and none of the Notch-related SNPs were significantly associated with susceptibility to HCC.

## Figures and Tables

**Table 1:** Demographic, laboratory and histopathology information on different study groups.

Characteristics	Controls	ALC	ALC + HCC	*p* Value
N of patients	141	132	128	
Age ^1^, years	61.42 ± 14.58	60.24 ± 6.27	61.38 ± 7.47	0.57
Sex ^2^				
m	125 (88.7%)	116 (87.9%)	117 (91.4%)	0.63
f	16 (11.3%)	16 (12.1%)	11 (8.6%)	
ALT (IU/L)^3^		23 (18.5–33)	32 (24–45)	2.4 × 10^−4^
AST (IU/L)^3^		47 (36–63)	50 (39–70)	0.92
Creatinine (µmol/L)^3^		91 (66–127)	79 (63–101)	0.011
Platelets (×10^9^/L)^3^		87 (66.5–136.5)	93 (69–125)	0.84
AFP (nmol/L)^3^	-	3.8 (2.9–5.35)	7.3 (4.3–20.7)	3.5 × 10^−8^
Tumor size (mm)			34.15 ± 19.84	-
Angioinvasion ^4^				
yes			45 (37.2%)	
no			76 (62.8%)	-
Milan criteria ^4^				
within			63 (52.1%)	
beyond			58 (47.9%)	-

^1^ mean ± standard deviation; *p* value was calculated by ANOVA and Bonferroni’s post-hoc comparisons test; ^2^ *p* value was calculated by chi squared test. ^3^ Median with interquartile range; *p* value was calculated by Mann-Whitney-U test. ^4^ Only for patients who received transplantation. AFP—alpha-fetoprotein; ALC—alcoholic liver cirrhosis; ALT—alanine aminotransferase; AST—aspartate transaminase.

**Table 2:** Single nucleotide polymorphism-associated odds ratio for cirrhosis occurrence.

Gene SNP	Model	Genotype	ALC		OR	*p*
			No	Yes		
*PNPLA3* rs738409	Codominant	CC	80 (56.7%)	82 (31.5%)	1.00	
		CG	52 (36.9%)	122 (46.9%)	2.29 (1.46–3.58)	4 × 10^−7^
		GG	9 (6.4%)	56 (21.5%)	6.07 (2.82–13.09)	
	Dominant	CC	80 (56.7%)	82 (31.5%)	1.00	
		CG + GG	61 (43.3%)	178 (68.5%)	2.85 (1.86–4.35)	5.8 × 10^−6^
	Recessive	CC + CG	132 (93.6%)	204 (78.5%)	1.00	
		GG	9 (6.4%)	56 (21.5%)	4.03 (1.93–8.41)	8.4 × 10^−^^5^
	Log additive				2.39 (1.73–3.32)	6.3 × 10^−8^
*EGF* rs4444903	Codominant	AA	49 (34.8%)	82 (31.5%)	1.00	
		AG	66 (46.8%)	133 (51.1%)	1.20 (0.76–1.91)	0.7
		GG	26 (18.4%)	45 (17.3%)	1.03 (0.57–1.88)	
	Dominant	AA	49 (34.8%)	82 (31.5%)	1.00	
		AG + GG	92 (65.2%)	178 (68.5%)	1.16 (0.75–1.78)	0.51
	Recessive	AA + AG	115 (81.6%)	215 (82.7%)	1.00	
		GG	26 (18.4%)	45 (17.3%)	0.93 (0.54–1.58)	0.78
	Log additive				1.04 (0.78–1.40)	0.77
*TM6SF2* rs58542926	Codominant	CC	126 (89.4%)	194 (74.6%)	1.00	
		CT	15 (10.6%)	61 (23.5%)	2.64 (1.44–4.85)	7.6 × 10^−4^
		TT	0 (0%)	5 (1.9%)	NA	NA
	Dominant	CC	126 (89.4%)	194 (74.6%)	1.00	
		CT + TT	15 (10.6%)	66 (25.4%)	2.86 (1.56–5.23)	4.4 × 10^−4^
	Recessive	CC + CT	141 (100%)	255 (98.1%)	1.00	
		TT	0 (0%)	5 (1.9%)	NA	NA
	Log additive				2.83 (1.58–5.07)	3 × 10^−4^
*MTHFR* rs1801133	Codominant	GG	58 (41.1%)	120 (46.1%)	1.00	
		AG	64 (45.4%)	114 (43.9%)	0.86 (0.56–1.33)	0.46
		AA	19 (13.5%)	26 (10%)	0.66 (0.34–1.29)	
	Dominant	GG	58 (41.1%)	120 (46.1%)	1.00	
		AG + AA	83 (58.9%)	140 (53.9%)	0.82 (0.54–1.23)	0.33
	Recessive	GG + AG	122 (86.5%)	234 (90%)	1.00	
		AA	19 (13.5%)	26 (10%)	0.71 (0.38–1.34)	0.3
	Log additive				0.83 (0.61–1.12)	0.23
*NOTCH1* rs3124591	Codominant	TT	47 (33.3%)	79 (30.4%)	1.00	
		CT	59 (41.8%)	127 (48.9%)	1.28 (0.80–2.06)	0.39
		CC	35 (24.8%)	54 (20.8%)	0.92 (0.53–1.60)	
	Dominant	TT	47 (33.3%)	79 (30.4%)	1.00	
		CT + CC	94 (66.7%)	181 (69.6%)	1.15 (0.74–1.78)	0.54
	Recessive	TT + CT	106 (75.2%)	206 (79.2%)	1.00	
		CC	35 (24.8%)	54 (20.8%)	0.79 (0.49–1.29)	0.35
	Log additive				0.98 (0.74–1.30)	0.88
*NOTCH3* rs1043996	Codominant	AA	71 (50.4%)	144 (55.4%)	1.00	
		AG	51 (36.2%)	101 (38.9%)	0.98 (0.63–1.52)	0.036 *^q^* ^> 0.05^
		GG	19 (13.5%)	15 (5.8%)	0.39 (0.19–0.81)	
	Dominant	AA	71 (50.4%)	144 (55.4%)	1.00	
		AG + GG	70 (49.6%)	116 (44.6%)	0.82 (0.54–1.23)	0.34
	Recessive	AA + AG	122 (86.5%)	245 (94.2%)	1.00	
		GG	19 (13.5%)	15 (5.8%)	0.39 (0.19–0.80)	0.01
	Log additive				0.74 (0.54–1.01)	0.061
*NOTCH3* rs1044116	Codominant	TT	84 (59.6%)	165 (63.5%)	1.00	
		CT	45 (31.9%)	84 (32.3%)	0.95 (0.61–1.49)	0.22
		CC	12 (8.5%)	11 (4.2%)	0.47 (0.20–1.10)	
	Dominant	TT	84 (59.6%)	165 (63.5%)	1.00	
		CT + CC	57 (40.4%)	95 (36.5%)	0.85 (0.56–1.29)	0.44
	Recessive	TT + CT	129 (91.5%)	249 (95.8%)	1.00	
		CC	12 (8.5%)	11 (4.2%)	0.47 (0.20–1.11)	0.086
	Log additive				0.80 (0.57–1.12)	0.20
*NOTCH4* rs422951	Codominant	CC	33 (23.4%)	71 (27.3%)	1.00	
		CT	75 (53.2%)	128 (49.2%)	0.79 (0.48–1.31)	0.66
		TT	33 (23.4%)	61 (23.5%)	0.86 (0.48–1.55)	
	Dominant	CC	33 (23.4%)	71 (27.3%)	1.00	
		CT + TT	108 (76.6%)	189 (72.7%)	0.81 (0.51–1.31)	0.39
	Recessive	CC + CT	108 (76.6%)	199 (76.5%)	1.00	
		TT	33 (23.4%)	61 (23.5%)	1.00 (0.62–1.63)	0.99
	Log additive				0.92 (0.69–1.24)	0.6

*^q^*^> 0.05^ The difference did not reach statistically significant levels after correction for multiple comparison. ALC—alcoholic liver cirrhosis; *EGF*—epidermal growth factor; *MTHFR*—methylenetetrahydrofolate reductase; NA—not analyzed; not meaningful due to low minor allele frequency (see limitations in the Discussion section); OR—odds ratio; *PNPLA3*—patatin-like phospholipase domain-containing protein 3; *TM6SF2*—transmembrane 6 superfamily member 2.

**Table 3:** Logistic regression analysis of predictors of cirrhosis development.

Variables in the Model ^1^	b	Wald	*p*	OR (95%CI)	Adjusted Model ^2^	
					*p*	OR (95%CI)
*PNPLA3* (rs738409)						
C/C—C/G	reference	-	-	1.00	-	1.00
G/G	1.47	14.2	1.6 × 10^−4^	4.21 (1.99–8.87)	1.7 × 10^−4^	4.20 (1.99–8.87)
*TM6SF2* (rs58542926)						
C/C	reference	-	-	1.00	-	1.00
C/T—T/T	1.06	11.5	0.001	2.89 (1.56–5.35)	0.001	2.89 (1.57–5.35)
*NOTCH3* (rs1043996)						
A/A—A/G	reference	-	-	1.00	-	1.00
GG	−0.82	4.75	0.029	0.44 (0.21–0.92)	0.025	0.43 (0.20–0.90)

^1^*PNPLA3*, *TM6SF2* and *NOTCH3* polymorphisms were included in the model. ^2^ Model additionally adjusted for sex and age. For each model b coefficient is shown with corresponding Wald statistic value, odds ratio with 95% confidence interval, and *p* value. OR—odds ratio; *PNPLA3*—patatin-like phospholipase domain-containing protein 3; *TM6SF2*—transmembrane 6 superfamily member 2.

**Table 4:** Single-nucleotide polymorphism-associated odds ratio for HCC occurrence.

Gene SNP	Model	Genotype	HCC		OR	*p*
			No	Yes		
*PNPLA3* rs738409	Codominant	CC	47 (35.6%)	35 (27.3%)	1.00	
		CG	68 (51.5%)	54 (42.2%)	1.07 (0.61–1.88)	0.002
		GG	17 (12.9%)	39 (30.5%)	3.08 (1.50–6.32)	
	Dominant	CC	47 (35.6%)	35 (27.3%)	1.00	
		CG + GG	85 (64.4%)	93 (72.7%)	1.47 (0.87–2.49)	0.15
	Recessive	CC + CG	115 (87.1%)	89 (69.5%)	1.00	
		GG	17 (12.9%)	39 (30.5%)	2.96 (1.57–5.58)	5.6 × 10^−4^
	Log additive				1.66 (1.17–2.35)	0.004
*EGF* rs4444903	Codominant	AA	51 (38.6%)	31 (24.2%)	1.00	
		AG	61 (46.2%)	72 (56.3%)	1.94 (1.11–3.41)	0.042 ^*q* > 0.05^
		GG	20 (15.2%)	25 (19.5%)	2.06 (0.98–4.30)	
	Dominant	AA	51 (38.6%)	31 (24.2%)	1.00	
		AG + GG	81 (61.4%)	97 (75.8%)	1.97 (1.15–3.36)	0.012
	Recessive	AA + AG	112 (84.8%)	103 (80.5%)	1.00	
		GG	20 (15.2%)	25 (19.5%)	1.36 (0.71–2.59)	0.35
	Log additive				1.50 (1.05–2.16)	0.026 ^*q* > 0.05^
*TM6SF2* rs58542926	Codominant	CC	100 (75.8%)	94 (73.4%)	1.00	
		CT	31 (23.5%)	30 (23.4%)	1.03 (0.58–1.83)	0.36
		TT	1 (0.8%)	4 (3.1%)	4.26 (0.47–38.72)	
	Dominant	CC	100 (75.8%)	94 (73.4%)	1.00	
		CT + TT	32 (24.2%)	34 (26.6%)	1.13 (0.65–1.98)	0.67
	Recessive	CC + CT	131 (99.2%)	124 (96.9%)	1.00	
		TT	1 (0.8%)	4 (3.1%)	4.23 (0.47–38.29)	0.15
	Log additive				1.22 (0.74–2.01)	0.44
*MTHFR* rs1801133	Codominant	GG	57 (43.2%)	63 (49.2%)	1.00	
		AG	61 (46.2%)	53 (41.4%)	0.79 (0.47–1.31)	0.62
		AA	14 (10.6%)	12 (9.4%)	0.78 (0.33–1.81)	
	Dominant	GG	57 (43.2%)	63 (49.2%)	1.00	
		AG + AA	75 (56.8%)	65 (50.8%)	0.78 (0.48–1.28)	0.33
	Recessive	GG + AG	118 (89.4%)	116 (90.6%)	1.00	
		AA	14 (10.6%)	12 (9.4%)	0.87 (0.39–1.96)	0.74
	Log additive				0.84 (0.58–1.22)	0.37
*NOTCH1* rs3124591	Codominant	TT	40 (30.3%)	39 (30.5%)	1.00	
		CT	63 (47.7%)	64 (50.0%)	1.04 (0.59–1.83)	0.88
		CC	29 (22.0%)	25 (19.5%)	0.88 (0.44–1.77)	
	Dominant	TT	40 (30.3%)	39 (30.5%)	1.00	
		CT + CC	92 (69.7%)	89 (69.5%)	0.99 (0.58–1.68)	0.98
	Recessive	TT + CT	103 (78.0%)	103 (80.5%)	1.00	
		CC	29 (22.0%)	25 (19.5%)	0.86 (0.47–1.57)	0.63
	Log additive				0.95 (0.67–1.34)	0.77
*NOTCH3* rs1043996	Codominant	AA	74 (56.1%)	70 (54.7%)	1.0	
		AG	54 (40.9%)	47 (36.7%)	0.92 (0.55–1.53)	0.14
		GG	4 (3%)	11 (8.6%)	2.91 (0.88–9.56)	
	Dominant	AA	74 (56.1%)	70 (54.7%)	1.00	
		AG + GG	58 (43.9%)	58 (45.3%)	1.06 (0.65–1.72)	0.82
	Recessive	AA + AG	128 (97%)	117 (91.4%)	1.00	
		GG	4 (3%)	11 (8.6%)	3.01 (0.93–9.71)	0.06
	Log additive				1.21 (0.81–1.81)	0.35
*NOTCH3* rs1044116	Codominant	TT	84 (63.6%)	81 (63.3%)	1.00	
		CT	45 (34.1%)	39 (30.5%)	0.90 (0.53–1.52)	0.25
		CC	3 (2.3%)	8 (6.2%)	2.77 (0.71–10.79)	
	Dominant	TT	84 (63.6%)	81 (63.3%)	1.00	
		CT + CC	48 (36.4%)	47 (36.7%)	1.02 (0.61–1.68)	0.95
	Recessive	TT + CT	129 (97.7%)	120 (93.8%)	1.00	
		CC	3 (2.3%)	8 (6.2%)	2.87 (0.74–11.06)	0.11
	Log additive				1.14 (0.75–1.75)	0.54
*NOTCH4* rs422951	Codominant	CC	40 (30.3%)	31 (24.2%)	1.0	
		CT	63 (47.7%)	65 (50.8%)	1.33 (0.74–2.39)	0.53
		TT	29 (22.0%)	32 (25.0%)	1.42 (0.72–2.83)	
	Dominant	CC	40 (30.3%)	31 (24.2%)	1.00	
		CT + TT	92 (69.7%)	97 (75.8%)	1.36 (0.79–2.36)	0.27
	Recessive	CC + CT	103 (78.0%)	96 (75.0%)	1.00	
		TT	29 (22.0%)	32 (25.0%)	1.18 (0.67–2.10)	0.56
	Log additive				1.20 (0.85–1.69)	0.3

*^q^*^> 0.05^ The difference did not reach statistically significant levels after correction for multiple comparison. *EGF*—epidermal growth factor; *MTHFR*—methylenetetrahydrofolate reductase; OR—odds ratio; *PNPLA3*—patatin-like phospholipase domain-containing protein 3; *TM6SF2*—transmembrane 6 superfamily member 2.

**Table 5:** Logistic regression analysis of predictors for hepatocellular carcinoma development.

Variables in the Model ^1^	b	Wald	*p*	OR (95%CI)	Adjusted Model ^2^	
					*p*	OR (95%CI)
*PNPLA3* (rs738409)						
C/C—C/G	reference	-	-	1.00	-	1.00
G/G	1.12	11.7	0.001	3.07 (1.61–5.85)	0.001	3.10 (1.62–5.92)
*EGF* (rs4444903)						
A/A	reference	-	-	1.00	-	1.00
A/G—G/G	0.72	6.57	0.010	2.06 (1.19–3.57)	0.013	2.01 (1.16–3.51)

^1^ *PNPLA3* and *EGF* polymorphisms were included in the model. ^2^ Model additionally adjusted for sex and age. For each model b coefficient is shown with corresponding Wald statistic value, odds ratio with 95% confidence interval, and *p* value. *EGF*—epidermal growth factor; OR—odds ratio; *PNPLA3*—patatin-like phospholipase domain-containing protein 3.

## Data Availability

Data are contained within the article. Any additional data are available from the corresponding author upon reasonable request.

## Appendix A

**Table A1:** Assay IDs and expected minor allele frequency for the selected single-nucleotide polymorphisms.

SNP ID	Gene	Assay ID	Expected MAF ^1^
rs738409 C>G	*PNPLA3*	C______7241_10	22.5%
rs4444903 A>G	*EGF*	C__27031637_30	39%
rs58542926 C>T	*TM6SF26*	C__89463510_10	7%
rs1801133 G>A	*MTHFR*	C___1202883_20	37%
rs3124591 T>C	*NOTCH1*	C____189059_10	50%
rs1043996 A>G	*NOTCH3*	C__22275447_10	31%
rs1044116 T>C	*NOTCH3*	C___7494184_10	24%
rs422951 C>T	*NOTCH4*	C___3293790_10	46%

^1^ from the data for the European population in the SNP database, available at: https://www.ncbi.nlm.nih.gov/snp/ (accessed on 14 August 2021). *EGF*—epidermal growth factor; MAF—minor allele frequency; *MTHFR*—methylenetetrahydrofolate reductase; *PNPLA3*—patatin-like phospholipase domain-containing protein 3; *TM6SF2*—transmembrane 6 superfamily member 2.
